# Material Characterization of Silicones for Additive Manufacturing

**DOI:** 10.3390/polym16172437

**Published:** 2024-08-28

**Authors:** Danka Katrakova-Krüger, Simon Öchsner, Ester S. B. Ferreira

**Affiliations:** 1 Materials Laboratory, Faculty of Computer Science and Engineering Science, TH Köln, Campus Gummersbach, Steinmüllerallee 1, 51643 Gummersbach, Germany; simon-oechsner@t-online.de; 2 Cologne Institute of Conservation Sciences, Faculty of Cultural Sciences, TH Köln, Campus Südstadt, Ubierring 40, 50678 Cologne, Germany; ester.ferreira@th-koeln.de

**Keywords:** additive manufacturing, silicone, material extrusion, multi-jet fusion, material properties, chemical analysis

## Abstract

Three-dimensional printing is ideally suited to produce unique and complex shapes. In this study, the material properties of polysiloxanes, commonly named silicones, produced additively by two different methods, namely, multi-jet fusion (MJF) and material extrusion (ME) with liquid printing heads, are investigated. The chemical composition was compared via Fourier-transform infrared spectroscopy, evolved gas analysis mass spectrometry, pyrolysis gas chromatography coupled to mass spectrometry, and thermogravimetry (TGA). Density and low-temperature flexibility, mechanical properties and crosslink distance via freezing point depression were measured before and after post-treatment at elevated temperatures. The results show significant differences in the chemical composition, material properties, as well as surface quality of the tested products produced by the two manufacturing routes. Chemical analysis indicates that the investigated MJF materials contain acrylate moieties, possibly isobornyl acrylate linking branches. The hardness of the MJF samples is associated with crosslinking density. In the ashes after TGA, traces of phosphorus were found, which could originate from initiators or catalysts of the curing process. The ME materials contain fillers, most probably silica, that differ in their amount. It is possible that silica also plays a role in the processing to stabilize the extrusion strand. For the harder material, a higher crosslink density was found, which was supported also by the other tested properties. The MJF samples have smooth surfaces, while the ME samples show grooved surface structures typical for the material extrusion process. Post-treatment did not improve the material properties. In the MJF samples, significant color changes were observed.

## 1. Introduction

Silicones show many unique properties, such as good thermal and chemical stability against oxidizing agents, as well as radiation [1]. They also offer good biocompatibility and a wide temperature application range. These properties are related to their Si-O bonds in combination with the organic methyl groups and crosslinking between the molecular chains. Silicones are used in many different application fields. This includes the medical sector, cosmetic industry, model and prototype construction, food industry, automotive and aerospace, as well as electrical engineering [2,3,4,5,6,7,8]. These sectors often require a high level of flexibility regarding the part shape. Additive manufacturing, also called 3D printing, offers this freedom in design. In recent years, research in the field of additive manufacturing has grown significantly in all material classes [9]. Many processes, devices, and specific materials were further developed and improved, including 3D printing of soft materials [10,11,12,13]. Nowadays, a couple of different 3D-printing processes for silicones are available on the market [14,15,16,17,18]. Two of them are addressed comparatively in this work.

**Figure 1:**
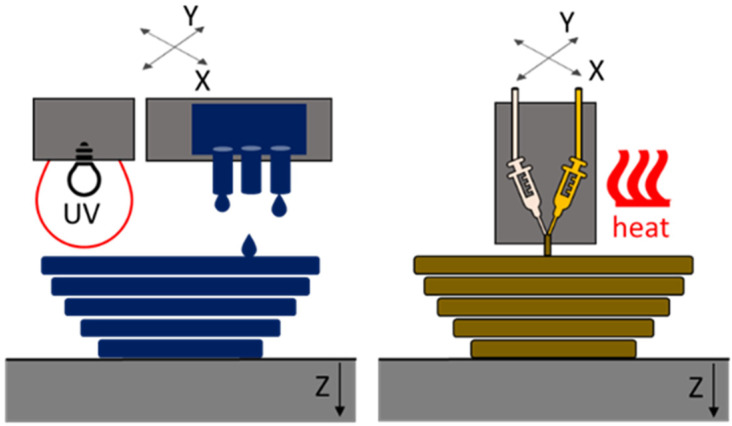
Working principles of the two researched processes: left: multi-jet fusion (MJF); right: material extrusion (ME).

The first manufacturing method considered in our study is based on the multi-jet fusion (MJF) process. Here, the curable material is deposited by multiple nozzles and is cured using UV or IR radiation [9,16]. In our case, UV exposure was used. The second manufacturing process is best fitted in the area of material extrusion (ME). It is comparable to the free filament fabrication (FFF) process. Similar processes are also known under the name Liquid Additive Manufacturing (LAM) [19,20]. These processes use Liquid Silicone Rubber (LSR), which is selectively deposited through a nozzle or syringe on the printing bed. Depending on the crosslinking mechanism, the silicone is crosslinked due to mixing with a second component (Room Temperature Vulcanization RTV-2) or exposure to heat or UV [14,15,21]. In our case, the process was heat-supported. In Figure 1, the working principles of the two processes are shown.

As each 3D-printing process requires the adaptation or development of suitable materials with specific properties, the aim of this study is to compare the material properties of the printed parts produced with the two different 3D-printing processes. This comparison may help to decide which technology will be best suited for a specific application based on achieved product quality. As the properties are strongly influenced by the degree of crosslinking and in the fast 3D printing, the crosslinking might not be completed, a thermal post-treatment (post-curing) was also performed to assess whether, through this, the properties might be improved, as it is common practice in standard silicone parts production [22].

## 2. Experimental

### 2.1. Materials

For each of the two printing processes, two different silicone materials were examined, which differ in their hardness. The investigated materials were labeled as follows:

- Two premixed silicones (RTV-1) manufactured with a multi-jet fusion process:
  - MJF-S (soft),
  - MJF-H (hard);
- Two RTV-2 silicones manufactured with a modified material extrusion process with liquid printing heads:
  - ME-S (soft),
  - ME-H (hard).

Sample plates with a thickness of 2 mm were used for the investigations. For the sample preparation, a die-cutter was used.

### 2.2. Methods

As a first step, the Shore A hardness was measured to quantify the soft and hard terms for the two processing routes. Fourier-transform infrared spectroscopy (FT-IR), evolved gas analysis mass spectrometry (EGA-MS), pyrolysis gas chromatography–mass spectrometry (Py-GC/MS), and thermogravimetry (TGA) were used to reveal the chemical composition of the materials and to understand how the hardness difference was achieved for each 3D-printing process. Physical properties like density, as well as glass transition temperature, were also determined. For the mechanical properties, tensile and tear resistance tests were performed. The crosslink density was addressed by crosslink distance measurement via freezing point depression and compression set tests.

The materials were then treated at two different temperatures to see if post-curing might be an option for better mechanical properties. Details on these methods are given below.

#### 2.2.1. Hardness

Shore A hardness was measured according to DIN ISO 48-4:2018 [23]. The mean value out of three measurements is given.

#### 2.2.2. Infrared Spectroscopy

FT-IR analysis was performed using a Nicolet iS10 from Thermo Fisher Scientific, Waltham, MA, USA in attenuated total reflection (ATR) mode.

#### 2.2.3. Evolved Gas Analysis Mass Spectrometry (EGA-MS) and Pyrolysis Gas Chromatography Mass Spectrometry (Py-GC/MS)

The chemical characterization of these performance-driven materials is challenging. Due to crosslinking, these polymers have a large molecular weight and are, thus, insoluble; therefore, multishot Py-GC/MS has been proven useful in their characterization [24]. EGA-MS was carried out to determine the optimal pyrolysis temperature and for general material identification.

Py-GC/MS analyses were performed using a multi-shot micro-furnace pyrolyzer, EGA/PY-3030D (Frontier Lab, Koriyama, Japan), coupled with a Finnigan Trace gas chromatography system with a split/splitless injection port and combined with a Thermo Fisher Scientific (Waltham, MA, USA) ISQ 7000 single quadrupole mass spectrometer.

The samples (300 × 300 × 300 µm^3^) were analyzed by single-shot pyrolysis and thermal desorption. The thermal desorption and pyrolysis temperatures were determined from the EGA-MS profile. The furnace-GC interface was set at 280 °C. The GC injector was operated in split mode, with a split ratio 1:80 and a temperature set to 260 °C. The chromatographic separation of pyrolysis products was performed on an Ultra ALLOY^®^ 5 capillary column HP-5MS (30 m × 0.25 mm i.d., 0.25 μm film thickness, Frontier Lab, Koriyama, Japan). The chromatographic conditions were 40 °C held for 5 min, followed by a 10 °C/min ramp to 300 °C, which was held for 10 min. The helium (purity 99.9995%) gas flow was set in constant flow mode at 1.2 mL/min. The mass spectrometer was operated in EI positive mode (70 eV, scanning *m*/*z* 35–750).

The mass spectrum interpretation was performed using the NIST 10.0 reference library (National Institute of Standards and Technology, Gaithersburg, MD, USA) and compared with the reference materials and the literature.

The instrumentation used for the EGA-MS analyses was the same as that used for the Py-GC/MS in a different instrumental configuration: the GC column was removed, and the pyrolyzer was coupled to the mass spectrometry system with a deactivated and uncoated stainless-steel transfer tube (UADTM-2.5N, 0.15 mm i.d. × 2.5 m length, Frontier Lab, Koriyama, Japan) kept at 300 °C, with an inlet temperature of 260 °C. The temperature program for the micro-furnace was an initial temperature of 100 °C, followed by a 20 °C/min ramp up to 700 °C. The furnace was kept at 700 °C for another 5 min. Analyses were performed under a helium flow (1 mL/min) with a 1:80 split ratio. The mass spectrometer was operated in EI positive mode (70 eV, scanning *m*/*z* 35–750).

#### 2.2.4. Thermogravimetry

For the thermogravimetry, the STA 409 PC from Netzsch-Gerätebau GmbH, Selb, Germany was used. Measurements were performed up to 700 °C with 10 K/min in an N_2_ atmosphere (10 mL/min). The ash was investigated via energy dispersive X-ray spectroscopy (EDS) in the scanning electron microscope (SEM) SU5000 from Hitachi High Tech Europe GmbH, Krefeld, Germany with EDS sensor X-Max 80 from Oxford Instruments GmbH, Wiesbaden, Germany.

#### 2.2.5. Density and Glass Transition

Density was measured with the buoyancy method in water, according to DIN EN ISO 1183-1 [25] at 23 °C. The glass transition temperature was determined by dynamic-mechanical analysis (DMA). For the measurements, DMA-3200 from TA Instruments, Eschborn, Germany with RSA G2 film fiber clamps was used. Evaluation of the temperature sweep between −140 and −50 °C with a frequency of 10 Hz, static elongation of 120%, and dynamic elongation of 0.04% was based on the maximum of the loss factor tanδ.

#### 2.2.6. Mechanical Testing

The mechanical properties were measured according to DIN 53,504 [26] using a Zwick Röll (Ulm, Germany) tabletop 10 kN universal testing machine. The tensile strength, elongation at break, stress at 100% elongation, and tear resistance were determined.

#### 2.2.7. Crosslink Density

To investigate the crosslink density the freezing point depression in cyclohexane, as used in [27], and the compression set were used. The compression set was determined for 168 h compression time for 25% deformation at room temperature.

#### 2.2.8. Post-Curing

The post-curing was performed in a convection oven. The temperatures and times of exposure were as follows for all samples: 150 °C for 4 h; 200 °C for 2 h; and 200 °C for 4 h. All analyses on the post-cured samples (except for DMA measurements and the tear resistance, which were not carried out) were performed in the same way as on the as-printed samples. The appearance of the samples after the post-curing was additionally examined optically with the spectrophotometer spectro-guide sphere gloss from BYK Additives & Instruments to document quantitatively any color changes. The L*a*b values were determined, and the color distance was calculated according to DIN EN ISO/CIE 1166-4 [28].

## 3. Results and Discussion

### 3.1. Shore A Hardness

**Figure 2:**
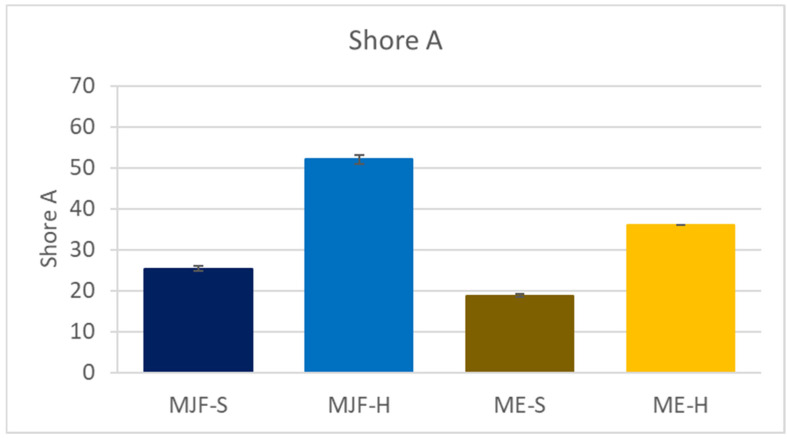
Shore A hardness of the investigated materials.

The Shore A hardness results are shown in Figure 2. The hardness of the MJF materials is higher than that of the corresponding ME materials. The following material characterization provides an explanation for the observed differences. 

### 3.2. Chemical Analyses

#### 3.2.1. Infrared Spectroscopy

**Figure 3:**
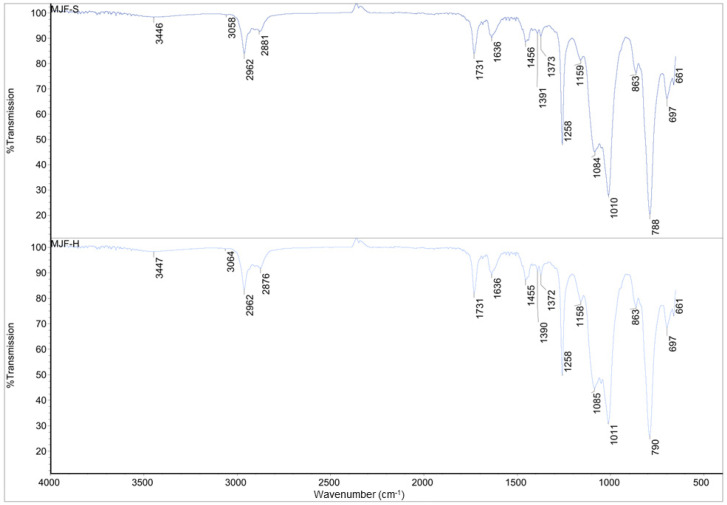
IR spectra for MJFS and MJF-H.

**Figure 4:**
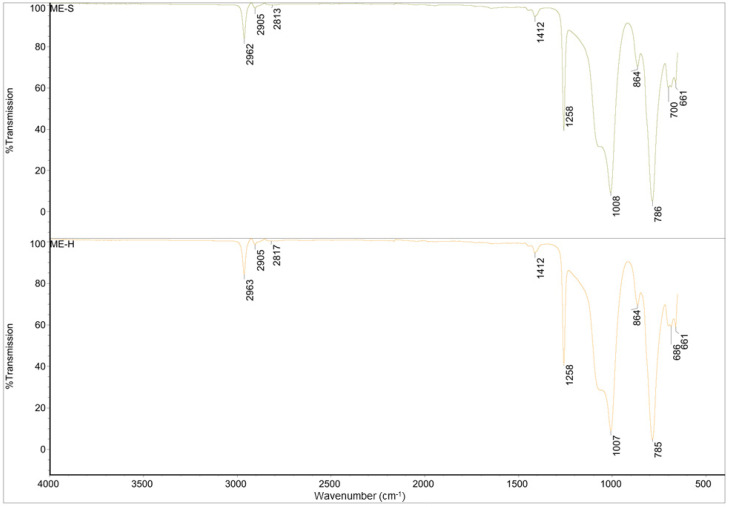
FTIR spectra of ME-S and ME-H.

The FT-IR spectra of the four materials are shown in Figure 3 and Figure 4.

The spectra of MJF-S and MJF-H are identical (correlation factor of 0.990), but they differ chemically from those of the ME materials. The small signals around 3450 cm^−1^ attributed to an -OH group and those near 1390 and 1370 cm^−1^ corresponding to -C-O- bonds indicate that the MJF silicones are terminated with -OH groups. Bands around 3060 cm^−1^ (asymmetric CH stretching -CH=CH_2_ group), 1730 cm^−1^ (-C=O), as well as at 1636 cm^−1^ (deformation vibration -CH=CH_2_ group) suggest the presence of acrylate functional groups.

For the spectra of ME-S and ME-H, it can be said that they are also identical and correspond to pure polydimethylsiloxane (PDMS) with a correlation factor of 0.995 [29,30]. The peaks around 2960 cm^−1^ and 2815 cm^−1^ are characteristic of the -CH_3_ symmetric and asymmetric stretching, and 1412 cm^−1^ can be attributed to -C-H bending vibrations in the methyl group. The peaks at 1258 cm^−1^ and 660–865 cm^−1^ are attributed to -Si-CH_3_ stretching and bending vibration, respectively.

#### 3.2.2. Evolved Gas Analysis Mass Spectrometry (EGA-MS) and Pyrolysis Gas Chromatography Mass Spectrometry (Py-GC/MS)

**Figure 5:**
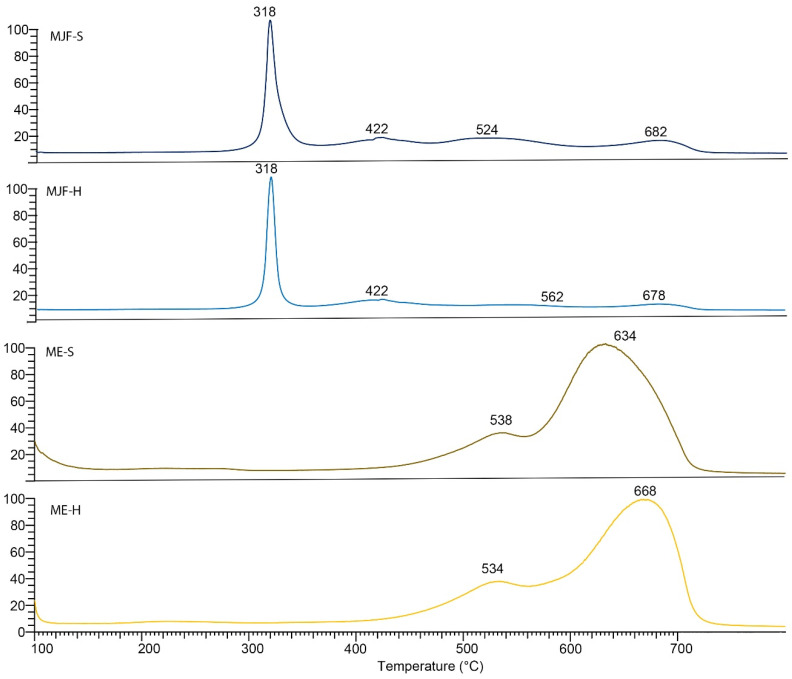
EGA-MS thermogram of MJF-S, MJF-H, ME-S, and ME-H samples.

**Figure 6:**
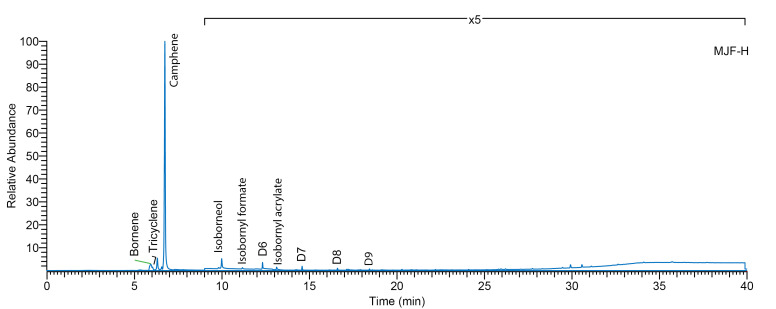
Thermal desorption (340 °C) gas chromatography–mass spectrometry (TD-GC/MS) of MJF-H sample.

The EGA-MS thermogram of MJF-S and MJF-H and the Total Ion Chromatogram (TIC) show a sharp event at 318 °C (Figure 5). Both the desorption temperature and the mass spectrum at this event (*m*/*z* 67, 77, 79, 91, 93, 107, 121, 136) are comparable to the reported degradation temperature and products of polyisobornyl acrylate [31]. The thermal desorption GC/MS (Figure 6) at a temperature of 340 °C shows the main component desorbed at that temperature from the MJF samples to be camphene (confirmed by mass spectral comparison and retention time relative to reference). Isobornyl acrylate (and related fragments) can be detected at very low levels. Isobornyl acrylate has been reported as a crosslinker in the UV curing of resins [32], but no published reference could be identified in the context of 3D-printed silicone elastomers. Since the uncured material was not analyzed, the interpretation remains unconfirmed; however, the data indicate that MJF materials are polydimethylsiloxane polymer and that the crosslinks between chains are achieved by UV activation of acrylates, possibly isobornyl acrylate.

The EGA-MS analysis of the ME-S and ME-H samples (Figure 5) is distinct and shows a bi-modal profile with maxima at 538 °C and 634 °C for ME-S and 534 °C and 668 °C for ME-H and average mass spectrum (*m*/*z* 73, 96, 133, 147, 191, 193, 207, 221, 267, 281, 325, 341, 399, 355, 415, 429) typical of PDMS [33]. The results suggest that ME-H has a higher thermal stability than ME-S since the thermal degradation event occurs at a higher temperature.

**Figure 7:**
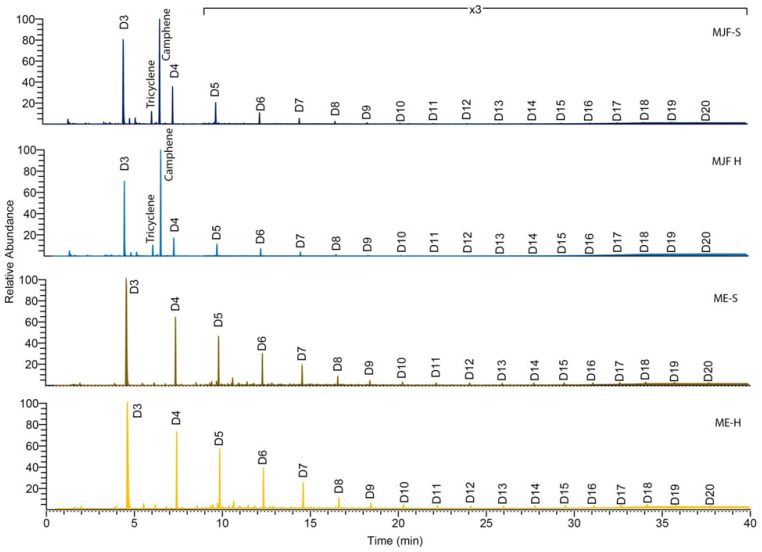
Pyrolysis (750 °C) gas chromatography–mass spectrometry (Py-GC/MS).

Py-GC/MS of all samples (Figure 7) results in a number of cyclic dimethylsiloxane pyrolysates (Dn where the n refers to the number of dimethylsiloxane units) typical of PDMS [33] which result from chain backbiting reactions [34]. Lewicki et al. [34] demonstrate that the distance between crosslinks is reflected in the pyrogram.

#### 3.2.3. Thermogravimetry

**Figure 8:**
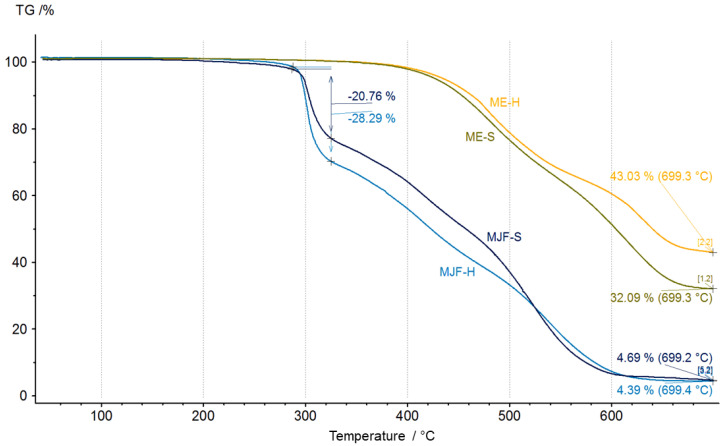
Thermogravimetric analysis thermogram of unaged materials.

Figure 8 shows the thermogravimetry results.

Differences between the materials are recognizable. The MJF materials show higher mass changes starting around 290 °C, which can be attributed to the release of camphene from the linking chains in the sample (equivalent to the event with a max at 318 °C in EGA-MS). The inorganic residue is, with ca. 5%, quite low for the MJF materials, so it is not likely that they contain mineral ingredients. Therefore, it is probable that the higher hardness is due to higher crosslinking. This is supported by the higher mass change measured at around 300 °C (28.29% vs. 20.76%) interpreted as related to the isobornyl acrylic fragments.

It is different for the ME materials. Significant mass changes are observed at temperatures above 400 °C, which was also seen in the EGA-MS. They show mineral residue, which may indicate the use of mineral filler. The chemical analysis identified only silicon and oxygen. Therefore, most probably, silica (SiO_2_) was used as a filler, which has been known since the 1950s as a good reinforcing agent in silicones and other rubber products [35,36,37,38]. It may also be used as a thixotropic agent to improve the processing behavior [39,40,41,42]. It has a positive effect on the stability of the extrusion strand. For the softer material, the mineral content is 31 ± 1%, whereas for the harder material, this value is 45 ± 3%. It indicates that the hardness was adjusted by the amount of filler and probably also by the higher crosslink density (see below).

**Figure 9:**
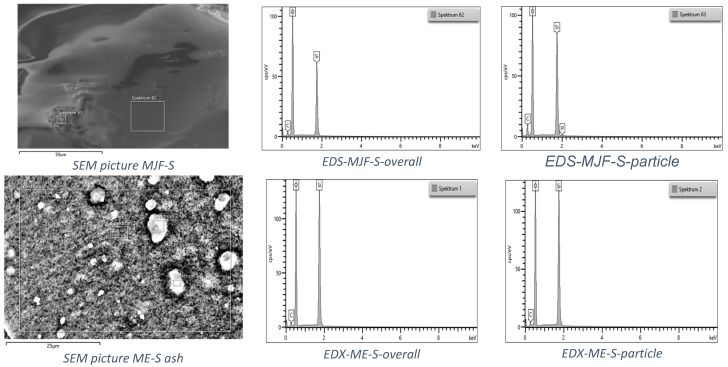
EDS spectra of the ash after TGA for the two soft materials.

The investigation of the ashes with SEM/EDS showed predominantly silicon and oxygen, as well as a small amount of carbon (only the soft materials are shown in Figure 9 as an example). In the MJF, ash traces of phosphorus were found, probably coming from initiators or catalysts for the crosslinking reaction [43]. Both harder materials show similar results and are, therefore, not given here.

### 3.3. Density and Glass Transition

The results for the density and the glass transition of the investigated materials are given in Table 1.

The density of the softer materials is marginally lower in both cases. It may be due to lower acrylic content for the MJF-S material and lower filler content for the ME-S material, as well as the lower crosslinking (see below).

The ME materials have a higher density compared to the MJF materials. This can be explained by the difference in the composition (ME contains fillers) as well as the manufacturing method. The glass transition for all four materials is from −115 °C to −119 °C in the typical range for silicones, being slightly higher for the ME materials, indicating lower mobility of the polymer chains, which can be due to the filler or higher crosslink density [44,45].

### 3.4. Mechanical Properties

**Figure 10:**
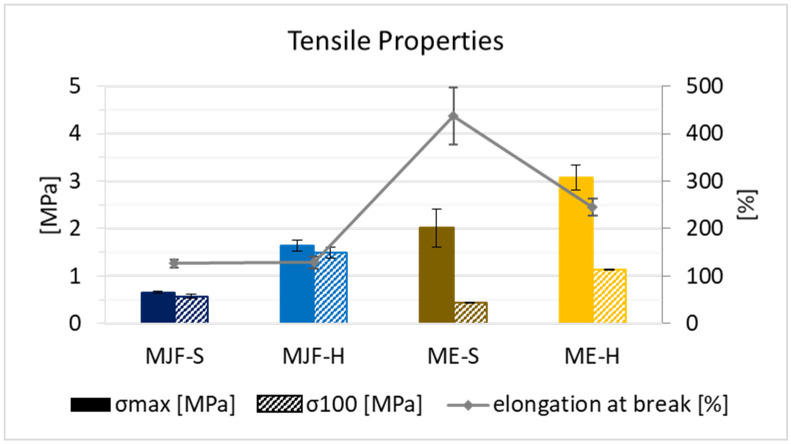
Tensile strength, stress at 100% elongation, and elongation at break for the four materials.

In terms of mechanical properties, it can be observed that the ME materials have higher tensile stress and elongation at break in direct comparison to the MJF material (see Figure 10). This can be explained by the presence of fillers, as they are often used exactly for the improvement in mechanical properties [35,36,37,38]. The tensile strength and stress at 100% elongation (often called modulus 100) are higher for the harder materials relative to the softer ones from both manufacturing routes. The elongation at break is identical for the MJF materials. Regarding the extruded materials (ME), it is lower for the harder material (ME-H). This can be due to the higher filler amount used to adjust the hardness. For the ultimate properties, a higher deviation is observed for the ME materials. This can also be due to the fillers and their not fully homogeneous distribution within the polymer. Interestingly, they are less stiff, which is seen in the lower stress levels at 100% elongation. The reason for that may be that the MJF materials are more thermoplastic due to the contained polar acrylic and hydroxylic groups.

The tear resistance results are summarized in Table 2. Generally, the harder materials show higher tear resistance, which can be due to the higher crosslinking. In addition, higher tear resistance values are observed for the ME materials compared to the MJF. This can be explained by the presence of fillers, which may deviate significantly and even stop cracks and slow down the crack propagation.

### 3.5. Crosslinking

The chemical network (crosslinks) decisively determines the properties of elastomers. Differences can be seen in the mechanical properties (hardness and tensile test, for example) and the compression set. The crosslink distance is a more direct measure of the crosslink density.

**Figure 11:**
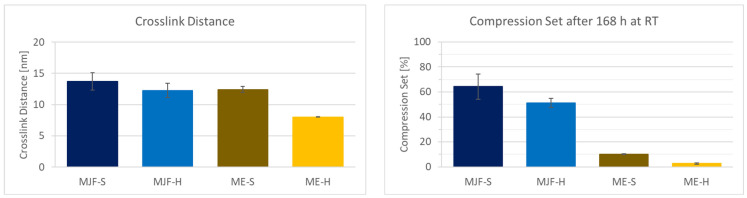
Crosslink distance and compression set of the four different materials.

Figure 11 shows the crosslink distance results obtained by freezing point depression and the compression set for the investigated materials. The hardness values can be correlated with the crosslink distance. For the two hard materials, as expected, the distance between the crosslinks is lower than for the soft materials, which means higher crosslink density. It is observed that the variation is lower for the ME materials, which speaks for more evenly distributed crosslinking. It must be mentioned that the presence of fillers in the ME materials shifts the values for the crosslink distance to lower values that are also dependent on the filler content. This is due to the fact that filler particles are between the macromolecular chains and reduce the solvent available volume. The compression set for the MJF materials is relatively high at approx. 50 and 65%, which indicates a lower elasticity of the material compared to the ME materials. These are in the range of approx. 10 and 4%, which is more common for silicones [46,47]. The higher compression set for the MFJ materials can be explained by the presence of the polar groups (acrylic and hydroxylic), resulting in a more thermoplastic behavior.

### 3.6. Post-Curing Study

Annealing with different conditions in terms of temperature and time was performed to see if the material properties could be improved due to eventual post-crosslinking. Another effect would be to ensure that condensation by-products are removed from the polymer before use [48].

#### 3.6.1. Color Changes

**Figure 12:**
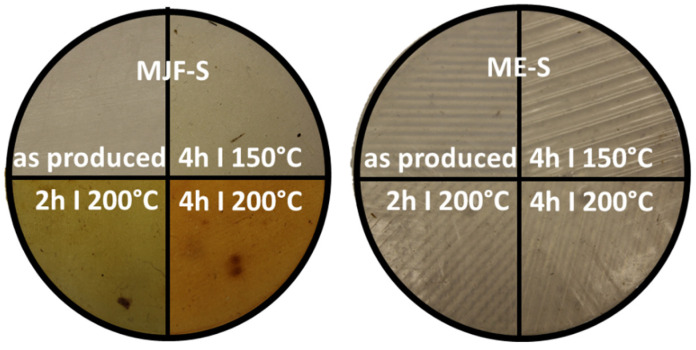
Optical changes for MJF-S and ME-S at the four different annealing stages (photography).

After the annealing study, more pronounced color changes for the MJF materials were observed. Figure 12 shows the appearance of the softer materials MJF-S and ME-S. The results were similar for the harder materials MJF-H and ME-H.

**Figure 13:**
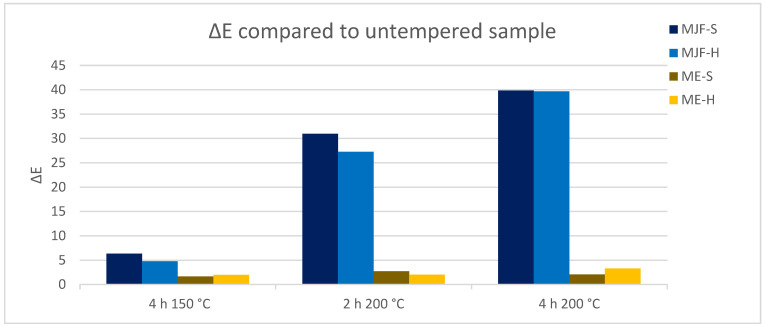
Color changes delta E due to tempering, measured with the L*a*b system.

The colorimeter measures the color of the sample with the parameters of the L*a*b system. With the help of the L*a*b values, the color distance Delta E was determined for the different annealing conditions. Figure 13 shows the change in perception of the individual samples over the annealing steps in relation to the unannealed sample. It is clearly visible that the MJF materials experience a higher color change, more pronounced at 200 °C, than the ME materials, which show only a hardly noticeable change over the tempering steps. The change in color was superficial and is possibly due to the oxidation of the isoborneol moieties of the linking chains at the specimen surface. EGA-MS and Py-GC/MS of the annealed MJF samples show a broader first event in EGA-MS and the presence of oxidized terpene species as pyrolysates in Py-GC/MS absent before the post-curing. It is more pronounced with higher temperature and time. In the FT-IR spectra, for both MJF materials, the bands around 3450 cm^−1^, at 1636 cm^−1^, and around 1390 cm^−1^ are missing after annealing, which can be explained with chemical reactions of the corresponding hydroxylic (-OH) and vinyl (-CH=CH_2_) groups. This is also supported by the thermogravimetry results showing earlier beginning (ca. 50 °C) of the acrylic decomposition and reduced mass change of around 4% for MJF-S. No changes are observed for the ME materials after the post-curing.

#### 3.6.2. Material Properties

**Figure 14:**
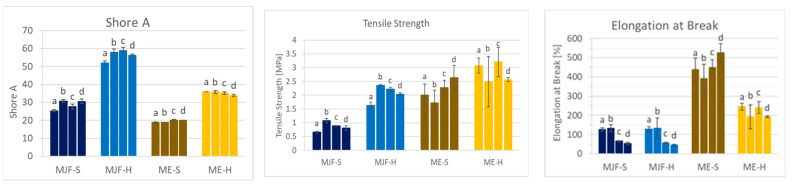
Shore A, tensile strength, and elongation at break after annealing: (a) as produced; (b) 4 h/150 °C; (c) 2 h/200 °C; (d) 4 h/200 °C.

**Figure 15:**
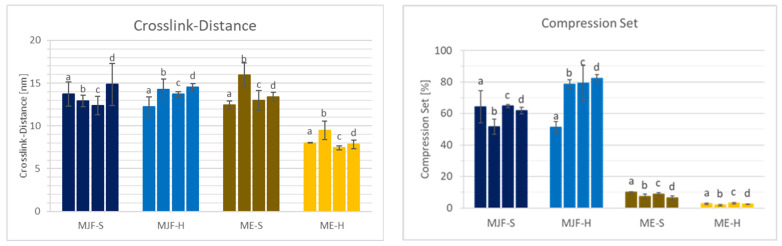
Crosslink distance and compression set after annealing: (a) as produced; (b) 4 h/150 °C; (c) 2 h/200 °C; (d) 4 h/200 °C.

The changes in the material properties compared to the as-printed samples are shown in Figure 14 and Figure 15.

For the softer MJF material, a slight post-curing effect is observed at 150 °C with increased hardness and tensile strength, preserving the elongation at break accompanied by reduced crosslink distance and lower compression set (comparison ‘a’ to ‘b’ in Figure 14 and Figure 15). The harder MJF material shows similar changes at 150 °C for the mechanical properties, but the crosslink distance and the compression set increased. A possible explanation is that the higher thermoplastic share in the harder material became predominant after the compression treatment. The ME materials behave differently from the MJF ones but similarly among themselves. At 150 °C, there is no hardness change, but lower tensile and elongation at break are observed in accordance with increased crosslink distance, implicating reversion as well. But, the lower compression set is contradictory. It may be due to increased filler (silica)–polymer bonding leading to higher elasticity as well as loss of the condensation products from the bonding reaction at the higher temperature.

Increasing the temperature to 200 °C (comparison ‘b’ to ‘d’ in Figure 14 and Figure 15) leads to a small decrease in the mechanical properties accompanied by increasing crosslink distance and compression set, meaning lower crosslink density resp. reversion for the MJF materials. The ME materials behave differently. While the hardness and the ultimate properties increase and the crosslink distance and the compression set decrease for the softer material, indicating additional crosslinking, the changes are very small for the harder material.

With increased annealing time (comparison of two, respectively, 4 h at 200 °C corresponding to ‘c’ and ‘d’ in Figure 14 and Figure 15) for the MJF materials, the same tendencies as for the higher temperature are observed but to a lower extent and with the difference that the hardness increases slightly for the softer material, whereas it decreases for the harder one. For the ME materials, differences are also observed. A slight decrease in the Shore A hardness is common for both, but the ultimate properties are higher for the softer material and lower for the harder one. The crosslink distance is slightly higher for both materials, and the compression set is slightly lower, which is contradictory at first. But it can be explained with additional chemical bonding between the silica and the polymer, bringing them closer together, resulting in higher crosslink distance (more room between the polymer chains) but increasing the elasticity at the same time, leading to lower compression set values.

Altogether, especially considering the deviation in the results, no clear advantage for the post-curing was found. The spreading increased in most cases, and for the MJF materials, color changes were clearly visible, which is rather disadvantageous for applications.

## 4. Conclusions and Outlook

The materials investigated in this study produced by different 3D-printing processes differ in their chemical composition and their material properties. The MJF materials contain acrylate moieties, possibly isobornyl acrylate linking branches. The difference in the hardness is due to higher crosslinking density for the harder material. In the ashes obtained after TGA, traces of phosphorus were found, which could originate from initiators or catalysts of the curing process. The ME materials contain fillers, most probably silica, that differ in their amount between hard and soft varieties. It is possible that silica may also play a role in the processing, being added sometimes for thixotropic behavior. The harder material shows not only higher filler content but also higher crosslink density. 

The density is respectively lower for the softer materials and the MJF materials than for the ME compared in this work. This can be explained by the presence of different filler amounts in the ME specimens and the higher crosslink density for the harder materials. The glass transition temperature is found to be lower for the MJF materials as the fillers in the investigated ME materials reduce the mobility of the polymer chains.

The ultimate properties of the considered ME materials are higher, but their stiffness is lower. This can be due to the presence of fillers, often added as reinforcing agents to improve the mechanical properties, whereas the acrylate content provides higher stiffness. The tear resistance was generally higher for the harder materials due to higher crosslinking density and higher for the ME materials, which can also be explained by the presence of fillers slowing down the crack propagation.

The harder materials for both processes show a higher level of crosslinking than the ME materials compared to the MJF. The compression set is unusually high for the MJF materials which may be due to the contained polar groups (hydroxylic and acrylic) leading to more thermoplastic behavior.

The surface of the materials differs greatly. The ME samples show grooved surface structures typical for the material extrusion process. The MJF samples, on the other hand, have smoother surfaces, which is due to the production of these via multi-jetting.

After annealing, color changes were observed for the MJF materials related to oxidation effects of the surface. Regarding the resulting properties, no clear advantage was seen. So, post-curing is not really needed for the additively manufactured parts with the materials considered in this study. Only the treatment at 150 °C for the MJF-S sample may be seen as favorable. But, the annealing time could be reduced. Generally, with higher temperatures and times, some hints of reversion were found. Mostly, the spreading of the results was higher after the heat treatment.

The investigations show that good quality components can be manufactured additively from silicones using the processes of multi-jet fusion and material extrusion. The MJF materials investigated would be particularly suitable for optical prototypes because of their good surface, whereas the ME materials would also be suitable for functional prototypes due to their good mechanical properties but are inferior with regard to their surface roughness. 

In further investigations, the processes that take place during annealing could be examined in detail to better understand the underlying mechanisms.

## Figures and Tables

**Table 1:** Density and glass transition temperature.

Properties	MJF-S	MJF-H	ME-S	ME-H
Density [g/cm^3^]	1.012	1.019	1.038	1.077
Glass transition temperature (Tg) [°C]	−118	−119	−115	−115

**Table 2:** Tear resistance of the investigated materials.

Properties	MJF-S	MJF-H	ME-S	ME-H
Tear resistance [kN/m]	1.8	5.2	7.2	8.4
Standard deviation	±0.06	±0.30	±0.79	±0.85

## Data Availability

The original contributions presented in the study are included in the article, further inquiries can be directed to the corresponding author.
